# Consumption a Curable and Preventable Disease

**Published:** 1903-12

**Authors:** 


Consumption a Curable and Preventable Disease. What a layman should know about it. By Lawrence F. Flick, M. D., Founder of the Pennsylvania Society for the Prevention of Tuberculosis. Duodecimo, 295 pages. Philadelphia: David McKay, Publisher. 1903. (Price, $1.00.)

This author has written an admirable little book for the layman, which every doctor will find helpful to him in instructing consumptives themselves and their families, in order to secure their intelligent coöperation in battling with this destructive infection. The nature of consumption, the prevalency and ravages of the disease, what predisposes to consumption, the manner in which consumption is spread, human and animal tuberculosis, the curability of the disease, the various factors entering into the treatment, and the questions of marriage and the care of offspring, are all considered.

The book is plainly written so that it will be understood by all; the views it sets forth are rational, hopeful and scientific, and for the good of suffering humanity it should have a wide circulation. M. J. F.

